# Revised System to Evaluate Measurement of Blood Chemistry Data From the Japanese National Health and Nutrition Survey and Prefectural Health and Nutrition Surveys

**DOI:** 10.2188/jea.JE20120032

**Published:** 2013-01-05

**Authors:** Masakazu Nakamura, Masahiko Kiyama, Akihiko Kitamura, Yoshinori Ishikawa, Shinichi Sato, Hiroyuki Noda, Nobuo Yoshiike

**Affiliations:** 1 National Cerebral and Cardiovascular Center, Department of Preventive Cardiology, CDC/CRMLN Lipid Reference Laboratory, Osaka, Japan; 1 国立循環器病研究センター予防健診部; 2 Osaka Center for Cancer and Cardiovascular Disease Prevention, Osaka, Japan; 2 大阪府立成人病センター集団検診第I部; 3 Chiba Prefectural Institute of Public Health, Chiba, Japan; 3 千葉県衛生研究所; 4 Public Health, Department of Social and Environmental Medicine, Graduate School of Medicine, Osaka University, Osaka, Japan; 4 大阪大学大学院医学系研究科医療経済産業政策学; 5 Cancer Control and Health Promotion Division, Health Service Bureau, Ministry of Health, Labour, and Welfare, Tokyo, Japan; 5 厚生労働省健康局がん対策・健康増進課; 6 Aomori University of Health and Welfare, Aomori, Japan; 6 青森県立保健大学健康科学部

**Keywords:** monitoring system, accuracy, precision, total error

## Abstract

**Background:**

We developed a monitoring system that uses total errors (TEs) to evaluate measurement of blood chemistry data from the National Health and Nutrition Survey (NHNS) and Prefectural Health and Nutrition Surveys (PHNS).

**Methods:**

Blood chemistry data from the NHNS and PHNS were analyzed by SRL, Inc., a commercial laboratory in Tokyo, Japan. Using accuracy and precision from external and internal quality controls, TEs were calculated for 14 blood chemistry items during the period 1999–2010. The acceptable range was defined as less than the upper 80% confidence limit for the median, the unacceptable range as more than twice the cut-off value of the acceptable range, and the borderline range as the interval between the acceptable and unacceptable ranges.

**Results:**

The TE upper limit for the acceptable and borderline ranges was 5.7% for total cholesterol (mg/dL), 9.9% for high-density lipoprotein cholesterol (mg/dL), 10.0% for low-density lipoprotein cholesterol (mg/dL), 10.4% for triglycerides (mg/dL), 6.6% for total protein (g/dL), 7.6% for albumin (g/dL), 10.8% for creatinine (mg/dL), 6.5% for glucose (mg/dL), 9.7% for γ-glutamyl transpeptidase (U/L), 7.7% for uric acid (mg/dL), 8.7% for urea nitrogen (mg/dL), 9.2% for aspartate aminotransferase (U/L), 9.5% for alanine aminotransferase (U/L), and 6.5% for hemoglobin A1c (%).

**Conclusions:**

This monitoring system was established to assist health professionals in evaluating the continuity and comparability of NHNS and PHNS blood chemistry data among survey years and areas and to prevent biased or incorrect conclusions.

## Abstract (ja)

**【背景】:**

わが国の国民健康・栄養調査と都道府県民健康・栄養調査で集計される血液化学検査成績を総合誤差の大きさから評価・判別するためのモニタリング・システムを再構築した。

**【方法】:**

国民健康・栄養調査の全部と一部の都道府県民健康・栄養調査の血液検体は、㈱SRL（東京都八王子市）で同一装置・同一精度管理体制のもとに委託分析されている。1999年から2010年までの10年間における血液化学検査14項目を対象に、SRLの外部精度管理成績から正確度を、内部精度管理成績から精密度を求め、2つの指標から総合誤差(Total Error、%)を計算した。総合誤差の許容範囲は10年間の中央値の信頼限界の80%以下に、非許容範囲はその2倍の値と規定し、許容範囲と非許容範囲の中間域をﾎﾞｰﾀﾞｰﾗｲﾝと規定した。判定は、血液化学検査項目の総合誤差が許容範囲に入る場合は使用可、ﾎﾞｰﾀﾞｰﾗｲﾝに入る場合は注意して使用可、非許容範囲に入る場合は利用することは望ましくないとする。

**【結果】:**

計算の結果、ﾎﾞｰﾀﾞｰﾗｲﾝを含む総合誤差の上限値は次の値となった。総コレステロール5.7%、HDLコレステロール9.9%、LDLコレステロール10.0%、トリグリセライド10.4%、総タンパク6.6%、アルブミン7.6%、クレアチニン10.8%、ブドウ糖6.5%、γ-GTP 9.7%、尿酸9.7%、尿酸7.7%、尿素窒素8.7%、GOT 9.2%、GPT 9.5%、HbA1c 6.5%であった。

**【結論】:**

医学研究者が、わが国の国民健康・栄養調査と都道府県民健康・栄養調査で集計された14項目の血液化学検査成績を利用して、その項目の経年的な継続性や地域比較の可能性を検討するに際し、非許容範囲に入る成績を使うことによって予期しない錯誤や誤った結論に到達するリスクを避けるために、総合誤差による3レベル（Acceptable、Borderline、Unacceptable）のモニタリング・システムを再構築した。

## INTRODUCTION

In November every year, the Japanese Ministry of Health, Labour, and Welfare conducts the National Health and Nutrition Survey (NHNS) in 300 unit areas. In addition, some local governments conduct an independent Prefectural Health and Nutrition Survey (PHNS) of extended samples, according to the procedures used for the NHNS. All blood samples collected in the NHNS, and some blood samples obtained in the PHNS, are analyzed by SRL Inc., a commercial laboratory in Tokyo, Japan, and measurements are performed using the same analytic system.

All measurement is subject to error. Errors are not always constant and can differ by survey year depending on variations in many factors, including the principles underlying the method, analytic instruments, reagents, calibrator, medical technologist, and other laboratory conditions.^1^^,^^2^ Even if the external and internal quality controls used at SRL are sound, measurement errors are inevitable.

The monitoring system described in this study outlines principles that can be used by physicians and other health professionals who are interested in the continuity and comparability among survey years, or in the statistical results for components of physical examinations, in the annual NHNS and PHNS reports. Using these principles, they can determine by themselves if the results after 2011 can be used, should be used with care, or cannot be recommended for use according to the newly established TE criteria, which are based on external and internal quality controls at SRL during the 12-year period 1999–2010. The criteria for TEs were developed for use in monitoring during 2011–2015 but not for evaluating past data. Because the results of the analysis of collected data are open to the public but information on analytic errors is not, we hoped to prevent researchers from reaching biased or incorrect conclusions in their evaluations.

In 2008, we reported tentative monitoring principles that could be used to compare blood chemistry data obtained by the NHNS.^3^ However, after 2008, more PHNS data became available, to allow for evaluation of local plans in Health Japan 21. In addition, the number of blood chemistry items in the NHNS varies and has tended to increase. Finally, the Metabolic Syndrome-Focused Health Checkups Program^4^ in Japan began throughout the country in 2008. Due to these developments, we decided to revise the 2008 monitoring system.

## METHODS

### Blood chemistry items

In this study, 14 blood chemistry items (method, unit of measure at SRL) were evaluated: total cholesterol (TC) (enzymatic, mg/dL), high-density lipoprotein cholesterol (HDL-C) (homogeneous, mg/dL), low-density lipoprotein cholesterol (LDL-C) (homogeneous, mg/dL), triglycerides (enzymatic, mg/dL), total protein (Biuret, g/dL), albumin (bromcresol green, g/dL), creatinine (enzymatic, mg/dL), glucose (enzymatic, mg/dL), γ-glutamyl transpeptidase (γ-GT, γ-GTP) (Japanese Committee for Clinical Laboratory Standards [JSCC] recommended method, U/L), uric acid (enzymatic, mg/dL), urea nitrogen (enzymatic, mg/dL), aspartate aminotransferase (AST, GOT) (JSCC recommended, U/L), alanine aminotransferase (ALT, GPT) (JSCC recommended, U/L), and hemoglobin A1c (HbA1c) (latex agglutination-turbidimetric immunoassay [LA], %).

### External and internal quality control

SRL participates in the External Quality Assessment of Clinical Laboratories (EQACL) program of the Japan Medical Association (JMA)^5^ and the Lipid Standardization Program of the US Centers for Disease Control and Prevention/Cholesterol Reference Method Laboratory Network (CDC/CRMLN). SRL also has an internal quality control system that uses 2 concentrations of quality-control materials.

### Accuracy

Regarding accuracy (%bias) in Table 2, the evaluation method described in the 2010 annual report on EQACL by the JMA^5^ was as follows: (1) values that deviate by 3 SDs or more from the center are removed, the mean and SD are obtained according to the measurement method used by the laboratories that participated in the survey, and the coefficient of variation (CV) is calculated according to the measurement method; (2) measurement methods are arranged in order of increasing CV; (3) measurement methods with a high rank in at least 80% of laboratories are selected; (4) the mean of data from laboratories using the measurement methods selected in the previous step is calculated, 1-way analysis of variance is used to calculate intra-method variation (expressed as SD), and a common CV is obtained; and (5) the common CV is corrected for the report unit width and a corrected common CV is obtained. Using both the adjusted mean obtained from this iterative truncation method and measurement values obtained by SRL, %bias according to samples was calculated and the mean of multiple %bias (accuracy) was calculated as an index of systematic error.^6^

**Table 2.** SRL performance based on JMA external quality assessment and SRL internal quality control system (unit, %)

Analyte	Performance	Measurement performance by SRL during observation period													Proposed TE Criteria			*Application to new data*		(For reference) CAP TE Criteria
		
		Year												Median (LL, UL of 80% CL)	Acceptable	Borderline	Unacceptable	*Performance* *in 2011*	*Evaluation by proposed* *TE criteria in 2011*	

		1999	2000	2001	2002	2003	2004	2005	2006	2007	2008	2009	2010							
Total cholesterol	Accuracy (%bias)	0.19	−0.48	0.27	0.34	−0.15	−0.06	0.13	−0.82	−1.31	−1.45	−0.82	−0.66	−0.32 (−0.74, 0.04)				*0.19*		
	Precision (CV%)	1.7	1.6	1.3	1.1	1.6	1.0	1.2	1.0	0.7	0.8	0.7	0.7	1.1 (0.9, 1.3)				*0.8*		
	Total Error (%)	3.6	3.6	2.7	2.5	3.3	2.1	2.4	2.7	2.7	3.0	2.2	2.0	2.7 (2.5, 2.9)	<2.9	2.9–5.7	≥5.8	*1.8*	*acceptable*	5.0
HDL cholesterol	Accuracy (%bias)	−0.19	−1.57	−1.09	1.60	0.02	−0.33	0.70	1.29	−2.89	−0.90	−0.17	−0.68	−0.26 (−0.79, −0.08)				−*2.00*		
	Precision (CV%)	2.4	1.8	1.6	2.1	2.0	1.5	1.6	2.3	1.5	1.8	1.3	1.7	1.8 (1.6, 1.9)				*1.7*		
	Total Error (%)	4.9	5.1	4.2	5.7	4.0	3.2	3.8	5.7	5.8	4.4	2.7	4.0	4.3 (4.0, 5.0)	<5.0	5.0–9.9	≥10.0	*5.3*	*fairly acceptable*	15.0
LDL cholesterol	Accuracy (%bias)	—	—	—	—	—	—	—	—	−0.39	1.95	−2.45	0.50	0.06 (−1.42, 1.23)				*0.63*		
	Precision (CV%)	—	—	—	—	—	—	—	—	1.2	2.0	0.9	1.4	1.3 (1.1, 1.7)				*1.1*		
	Total Error (%)	—	—	—	—	—	—	—	—	2.7	5.9	4.2	3.2	3.7 (3.0, 5.0)	<5.0	5.0–10.0	≥10.1	*2.8*	*acceptable*	15.0
Triglycerides	Accuracy (%bias)	1.91	−0.58	−1.34	0.37	1.56	−0.12	−0.36	0.00	−0.97	−1.10	−1.86	−1.67	−0.47 (−1.04, −0.06)				−*0.18*		
	Precision (CV%)	1.8	2.3	2.4	2.6	2.3	1.5	1.4	2.3	1.0	1.0	1.1	1.2	1.7 (1.3, 2.3)				*1.6*		
	Total Error (%)	5.5	5.2	6.1	5.5	6.2	3.0	3.1	4.6	2.9	3.1	4.0	4.0	4.3 (3.6, 5.3)	<5.3	5.3–10.4	≥10.5	*4.4*	*acceptable*	12.5
Total protein	Accuracy (%bias)	−0.27	−0.12	0.46	−0.24	−0.14	−0.28	0.19	−0.07	−0.39	1.59	−0.58	1.78	−0.13 (−0.26, 0.06)				*3.21*		
	Precision (CV%)	1.4	1.0	0.9	1.5	2.0	1.6	1.4	1.5	1.5	1.6	1.0	1.3	1.5 (1.4, 1.5)				*1.3*		
	Total Error (%)	3.0	2.1	2.2	3.2	4.1	3.4	2.9	3.0	3.3	4.7	2.5	4.3	3.1 (3.0, 3.4)	<3.4	3.4–6.6	≥6.7	*5.8*	*fairly acceptable*	5.0
Albumin	Accuracy (%bias)	−2.43	−0.75	0.45	−1.12	0.64	0.12	−0.06	0.11	1.05	−0.28	−1.14	0.46	0.03 (−0.52, 0.29)				*5.19*		
	Precision (CV%)	1.7	1.3	2.0	1.8	1.9	1.2	1.6	1.1	0.9	1.2	1.0	1.2	1.3 (1.2, 1.6)				*1.0*		
	Total Error (%)	5.8	3.3	4.4	4.6	4.4	2.5	3.2	2.3	2.8	2.6	3.1	2.8	3.1 (2.8, 3.8)	<3.8	3.8–7.6	≥7.7	*7.1*	*fairly acceptable*	5.0
Creatinine	Accuracy (%bias)	−2.24	1.93	−0.08	−0.34	0.15	0.19	−0.76	−0.55	−0.76	−1.25	−0.54	−4.18	−0.55 (−0.76, −0.21)				−*2.77*		
	Precision (CV%)	1.5	2.6	3.7	2.0	1.9	2.3	1.8	2.3	1.7	2.3	1.3	1.8	2.0 (1.8, 2.3)				*1.7*		
	Total Error (%)	5.1	7.1	7.2	4.3	3.9	4.8	4.3	5.0	4.1	5.8	3.1	7.7	4.9 (4.3, 5.5)	<5.5	5.5–10.8	≥10.9	*6.1*	*fairly acceptable*	7.5
Glucose	Accuracy (%bias)	0.42	−0.58	−0.39	−0.31	0.17	−0.06	0.76	0.53	−0.83	−0.04	0.01	−0.74	−0.05 (−0.35, 0.09)				−*0.47*		
	Precision (CV%)	1.4	1.0	1.7	1.2	1.4	1.4	1.4	1.5	1.5	0.8	0.8	1.0	1.4 (0.8, 0.8)				*1.1*		
	Total Error (%)	3.1	2.5	3.7	2.7	3.0	2.7	3.5	3.5	3.8	1.6	1.6	2.7	2.9 (2.7, 3.3)	<3.3	3.3–6.5	≥6.6	*2.6*	*acceptable*	5.0
γ-GT (γ-GTP)	Accuracy (%bias)	0.74	−0.01	−0.24	0.82	0.37	−0.13	−0.48	−0.83	−1.50	0.45	−0.75	−1.04	−0.19 (−0.62, 0.18)				−*1.39*		
	Precision (CV%)	1.8	1.8	1.6	1.7	2.3	1.3	2.0	2.1	1.9	2.0	2.5	2.1	2.0 (1.8, 2.1)				*1.8*		
	Total Error (%)	4.2	3.5	3.4	4.2	4.8	2.7	4.4	5.0	5.2	4.4	5.7	5.2	4.4 (4.2, 4.9)	<4.9	4.9–9.7	≥9.8	*4.9*	*acceptable*	7.5
Uric acid	Accuracy (%bias)	0.21	−0.59	−0.43	0.25	−0.26	0.81	−0.44	0.88	−0.44	−0.56	0.31	1.26	−0.03 (−0.44, 0.28)				*1.11*		
	Precision (CV%)	2.1	2.1	1.4	1.5	1.4	1.4	1.8	1.5	1.6	1.1	1.3	1.6	1.5 (1.1, 1.1)				*1.1*		
	Total Error (%)	4.4	4.8	3.2	3.2	3.1	3.6	4.0	3.8	3.6	2.7	2.9	4.4	3.6 (3.2, 3.9)	<3.9	3.9–7.7	≥7.8	*3.3*	*acceptable*	8.5
Urea nitrogen	Accuracy (%bias)	−1.69	0.16	0.25	1.74	−0.17	0.75	−0.33	0.69	−2.86	—	—	1.58	0.21 (−0.25, 0.69)				*not assayed*		
	Precision (CV%)	1.3	1.2	1.2	1.7	1.8	1.1	1.9	1.4	1.5	—	—	1.5	1.5 (1.3, 1.6)				*not assayed*		
	Total Error (%)	4.3	2.6	2.7	5.1	3.7	3.0	4.1	3.4	5.8	—	—	4.5	3.9 (3.3, 4.4)	<4.4	4.4–8.7	≥8.8	*not assayed*		4.5
AST (GOT)	Accuracy (%bias)	3.03	−0.43	0.21	−0.07	1.37	0.59	−0.60	0.25	−1.25	0.51	0.71	0.64	0.38 (0.07, 0.62)				−*0.37*		
	Precision (CV%)	1.7	1.8	1.3	1.1	2.1	1.4	1.9	1.5	2.2	1.5	1.6	2.2	1.7 (1.5, 1.9)				*1.8*		
	Total Error (%)	6.3	4.0	2.7	2.3	5.5	3.4	4.4	3.3	5.6	3.5	3.8	5.0	3.9 (3.4, 4.6)	<4.6	4.6–9.2	≥9.3	*3.9*	*acceptable*	10.0
ALT (GPT)	Accuracy (%bias)	2.81	−0.22	0.38	−1.43	−0.08	1.48	1.06	−0.64	−1.47	0.95	0.88	0.37	0.38 (−0.15, 0.92)				−*1.12*		
	Precision (CV%)	1.4	1.7	1.4	1.4	2.3	1.5	2.3	2.2	2.2	1.6	1.8	2.2	1.8 (1.6, 2.2)				*2.3*		
	Total Error (%)	5.5	3.6	3.2	4.2	4.5	4.4	5.5	4.9	5.8	4.1	4.4	4.7	4.5 (4.3, 4.8)	<4.8	4.8–9.5	≥9.6	*5.6*	*fairly acceptable*	10.0
HbA_1_c	Accuracy (%bias)	—	—	−0.39	0.52	0.01	2.25	1.01	1.28	−0.34	−1.08	−0.14	−0.26	−0.07 (−0.30, 0.52)				*0.12*		
	Precision (CV%)	—	—	1.1	1.1	1.0	1.2	1.1	1.0	1.4	1.2	1.4	1.6	1.2 (1.1, 1.3)				*2.0*		
	Total Error (%)	—	—	2.5	2.7	2.0	4.6	3.2	3.2	3.1	3.4	2.9	3.4	3.1 (2.8, 3.3)	<3.3	3.3–6.5	≥6.6	*4.0*	*fairly acceptable*	

Accuracy as an index of systematic error is expressed as %bias calculated based on JMA criteria.

Precision as an index of random error is expressed as CV calculated from SRL internal quality control data.

Total error is calculated as the sum of accuracy and precision, ie, absolute value of %bias + 1.96 × CV.

Abbreviations: JMA, Japan Medical Association; CAP, College of American Pathologists; TE, total error; LL, lower limit; UL, upper limit; CL, confidence limit; HDL, high-density lipoprotein; LDL, low-density lipoprotein; γ-GT (γ-GTP), γ-glutamyl transpeptidase; AST (GOT), aspartate aminotransferase; ALT (GPT), alanine aminotransferase; HbA1c, hemoglobin A1c.

### Precision

Regarding precision (CV%) in Table 2, SD described in the EQACL represents dispersion in all participants, not the precision of measurement by SRL. Therefore, we were given data on the assayed values for 2 concentrations of internal quality control sera that were collected during a 1-month period, including values in November every year, randomly sampled 1 measurement value/day (*n* = 1) for 20 days, after which we calculated CV from the mean value and SD as an index of random error.^7^

### Total error and relevant criteria

Subsequently, TE was calculated from accuracy and precision. Regarding total error (%) in Table 2, the equation used was “accuracy (absolute value of %bias) + precision (1.96 × CV)”, which is used by the US National Cholesterol Education Program (NCEP) and the Lipid Standardization Program by CDC/CRMLN.^6^ The acceptable range of TE for each blood chemistry item was defined as less than the upper 80% confidence limit for the median of the 12-year period, as calculated by the nonparametric Bootstrap method (BC_a_ method).^8^^–^^10^ Bootstrap method analyses were conducted using SAS, version 13 (SAS Institute, Inc., Cary, NC, USA). The unacceptable range was defined as more than twice the cut-off value of the acceptable range, based on evaluation criteria adopted by the US College of American Pathologists (CAP).^11^ The interval between the acceptable and unacceptable ranges was classified as the borderline range. Thus, using these TE criteria, we have created a 3-level assessment of test performance.

### Use in evaluating performance in 2011

We collected the results of EQACL evaluations and SRL internal quality control data in 2011 and attempted to evaluate SRL test performance in 2011 using the proposed TE criteria.

### Criteria for CDC/CRMLN lipid standardization

To evaluate lipid measurement, the following NCEP criteria were used: TC—accuracy within 3% of target value for CDC/CRMLN reference measurement procedure, precision as CV of 3% or less, and TE of 9% or less; HDL-C—accuracy within 5% of target value, precision as CV 4% or less, and TE of 13% or less; LDL-C—accuracy within 4% of target value, precision as CV of 4% or less, and TE of 12% or less.^12^

### Implementation survey for PHNS

In 2007, our study group surveyed prefectural governments regarding implementation of their PHNS, including dietary intake surveys and blood examination, and collected additional data on the number of blood samples they entrusted to SRL for analysis in 2011.^13^

## RESULTS

Table 1 shows annual changes in blood chemistry items measured and number of analyzed NHNS samples assayed at SRL during 1999–2010. Items measured every year since 1999 were TC, HDL-C, triglycerides, total protein, and glucose. LDL-C, albumin, creatinine, and HbA1c were recently added to these 5 items. Other items, such as γ-GT (γ-GTP), uric acid, urea nitrogen, AST (GOT), and ALT (GPT), have been measured infrequently. The average number of assayed samples in the NHNS was 4704 during 1999–2010.

**Table 1.** Annual changes in numbers of assayed samples and blood chemistry items in the National Health and Nutrition Survey in Japan

Analyte	Year												*Application* *in 2011*

	1999	2000	2001	2002	2003	2004	2005	2006	2007	2008	2009	2010	
No. of assayed samples	5492	5743	5592	5413	5327	3921	3877	4319	4020	4517	4300	3930	*3515*

Total cholesterol	○	○	○	○	○	○	○	○	○	○	○	○	○
HDL cholesterol	○	○	○	○	○	○	○	○	○	○	○	○	○
LDL cholesterol	—	—	—	—	—	—	—	—	○	○	○	○	○
Triglycerides	○	○	○	○	○	○	○	○	○	○	○	○	○
Total protein	○	○	○	○	○	○	○	○	○	○	○	○	○
Albumin	—	—	—	—	○	○	○	○	○	○	○	○	○
Creatinine	—	○	—	—	—	—	—	—	—	○	○	○	○
Glucose	○	○	○	○	○	○	○	○	○	○	○	○	○
γ-GT (γ-GTP)	—	○	—	—	—	—	—	—	—	—	—	○	○
Uric acid	—	○	—	—	—	—	—	—	—	—	—	○	○
Urea nitrogen	—	○	—	—	—	—	—	—	—	—	—	—	—
AST (GOT)	—	—	—	—	—	—	—	—	—	—	—	○	○
ALT (GPT)	—	—	—	—	—	—	—	—	—	—	—	○	○
HbA1c	—	—	—	○	○	○	○	○	○	○	○	○	○

White circles show blood chemistry items assayed in the corresponding year.

Abbreviations: HDL, high-density lipoprotein; LDL, low-density lipoprotein; γ-GT (γ-GTP), γ-glutamyl transpeptidase; AST (GOT), aspartate aminotransferase; ALT (GPT), alanine aminotransferase; HbA1c, hemoglobin A1c.

Table 2 shows measurement performance at SRL, based on the EQACL of the JMA. On the basis of these calculations, criteria for acceptable, borderline, and unacceptable ranges were established, as shown in the column labeled Proposed TE Criteria.^10^ The upper limit of TE in the new acceptable and borderline ranges for each item was 5.7% for TC, 9.9% for HDL-C, 10.0% for LDL-C, 10.4% for triglycerides, 6.6% for total protein, 7.6% for albumin, 10.8% for creatinine, 6.5% for glucose, 9.7% for γ-GT (γ-GTP), 7.7% for uric acid, 8.7% for urea nitrogen, 9.2% for AST (GOT), 9.5% for ALT (GPT), and 6.5% for HbA_1C_. Concerning the acceptable TE range, 50% of the evaluation limits (1 side) of the CAP evaluation criteria, which are widely used worldwide, was adopted and is shown as a reference in the column labeled CAP TE in Table 2.^11^ TE criteria for HbA_1_c were not established in the CAP survey. Although the acceptable range for γ-GT (γ-GTP) is expressed as SD in the CAP evaluation criteria, 7.5% was used as the corresponding value.

A 2007 implementation survey showed that 25 (53.2%) of the 47 prefectures in Japan independently performed blood examinations. Blood examinations were entrusted to SRL by 21 of the 25 prefectures and to a local laboratory by the other 4. A total of 15 096 samples from the 21 prefectures were analyzed by SRL. This number was 3.2 times the mean sample number (4704) of the NHNS (Table 1). Additionally, according to the 2011 survey, 20 (42.6%) of the 47 prefectures performed blood examinations.

Blood examinations were entrusted to SRL by 15 of the 20 prefectures and to a local laboratory by the other 5. A total of 7063 samples from the 15 prefectures were analyzed by SRL. This number was 1.5 times the average sample number of the NHNS (Table 1). The survey of the current situation in each prefecture was not conducted systematically, and measurement items are different for each prefecture.

In 2011, urea nitrogen was not assayed in the NHNS or PHNS; thus, there was a total of 13 items. When TE was calculated for each SRL item in 2011 to establish proposed TE criteria, the evaluation was acceptable for 7 items (53.8%)—TC, LDL-C, triglycerides, glucose, γ-GT (γ-GTP), uric acid, and AST (GOT)—and borderline for 6 items (46.2%), namely, HDL-C, total protein, albumin, creatinine, ALT (GPT), and HbA_1_c. No item was evaluated as unacceptable (Table 2).

Table 3 shows the measurement performance of SRL for TC, HDL-C, and LDL-C, based on the criteria of the Lipid Standardization Program by CDC/CRMLN. In each standardization year, performance satisfied the CDC/CRMLN criteria for clinical laboratories.

**Table 3.** SRL performance based on CDC/CRMLN Lipid Standardization Program (unit, %)

Analyte	Performance	CDC Criteria	Year												Average	SD

			1999	2000	2001	2002	2003	2004	2005	2006	2007	2008	2009	2010		
Total cholesterol	Accuracy (%bias)	±3.0	0.00	−1.30	0.00	−0.90	0.30	−0.10	−0.90	−0.90	−0.90	−0.30	−0.50	0.10	−0.45	0.52
	Precision (CV%)	3.0	0.5	0.6	0.6	0.5	0.5	0.6	0.4	0.4	0.4	0.5	0.4	0.3	0.48	0.10
	Total Error (%)	9.0	1.0	2.5	1.2	1.9	1.3	1.4	1.7	1.7	1.7	1.3	1.3	0.8	1.48	0.45
HDL cholesterol	Accuracy (%bias)	±5.0	0.70	0.70	2.00	2.00	1.00	1.00	1.20	1.20	1.20	−1.00	0.00	0.00	0.83	0.85
	Precision (CV%)	4.0	1.0	1.0	1.3	1.3	1.7	1.7	1.1	1.1	1.1	1.0	0.7	0.7	1.14	0.32
	Total Error (%)	13.0	2.7	2.7	4.6	4.6	4.4	4.4	3.4	3.4	3.4	3.0	1.4	1.4	3.28	1.12
LDL cholesterol	Accuracy (%bias)	±4.0				−0.60	−0.60	−0.70	−0.70	0.30	0.30	1.70	−1.40	−1.40	−0.34	0.98
	Precision (CV%)	4.0				1.2	1.2	0.7	0.7	0.4	0.4	0.6	0.6	0.6	0.71	0.30
	Total Error (%)	12.0				3.0	3.0	2.1	2.1	1.1	1.1	2.9	2.6	2.6	2.28	0.75

Accuracy as an index of systematic error is expressed as %bias calculated based on CDC criteria.

Precision as an index of random error is expressed as CV calculated based on lipid standardization criteria of CDC.

Total error is calculated as the sum of accuracy and precision, ie, absolute value of %bias + 1.96 × CV.

Abbreviations: CDC, Centers for Disease Control and Prevention; CRMLN, Cholesterol Reference Method Laboratory Network; HDL, high-density lipoprotein; LDL, low-density lipoprotein.

## DISCUSSION

In standardization—the most advanced system of quality control assessment—target values are obtained by using globally accepted definitive or reference measurement procedures. However, in the EQACL, measurement values are collected from all participants and, after statistical analysis, adjusted mean values are obtained and used as an index of accuracy. A similar data processing method is used in external quality control assurance programs in Western countries.^14^^,^^15^ This method statistically excludes extreme outliers and misreports, which improves the reliability of adjusted mean values as indices of accuracy. Such adjusted means do not represent physicochemical accuracy, as such, but are often used for practical purposes as consensus values in clinical surveys. Consensus values are often used as a substitute for accuracy when there is no established reference method, or when a reference method exists but is not used due to its complexity or technical difficulty. In this respect, we have no objection to the use of consensus values at many laboratories, such as those derived from approximately 3000 participants in the EQACL of the JMA.^5^

The sources of error in measured values include changes in: the underlying principles of the measurement method, analytic devices, sample status (fresh, frozen), reagents or reagent reactivity, calibrators and their value assignments, the skill of analytical technologists, and other laboratory conditions.^1^^,^^2^^,^^5^^,^^6^ Measurement error can result in clinical examination-derived discontinuities with previously obtained results in surveys (such as retrospective case-control studies), which could markedly affect annual follow-up. In this study, we conducted detailed follow-up surveys of these factors to avoid discontinuities derived from clinical examinations. A disadvantage of using the mean value of an external quality assessment as an index of accuracy is that the method routinely used during each period has a direct influence on measurement values. For example, when an analytic method based on new measurement principles is developed and adopted at clinical laboratories, due to convenience and/or cost and time savings, changes in mean value are sometimes observed along with analytic errors.

Case 1: The routine analytic method for HDL-C changed from a precipitation method using polyanions and cations to a homogeneous method using detergent or surfactant. The new method has been adopted by many laboratories, and age-related changes in mean HDL-C values have been reported since the switch. In this former case, changes in mean HDL-C values were observed and, as a consequence, analytic errors change.^16^^–^^19^

Case 2: There has been increasing demand for more-precise creatinine analysis for people with diabetes mellitus and renal disorders, and the calibrator is changing from the old, water-soluble standard to a new serum-based reference material with high accuracy, as confirmed by gas chromatography/isotope dilution/mass spectrometry. Additionally, in many laboratories the creatinine method has changed from the classic Jaffe method to newly developed enzymatic methods. Changes in mean creatinine values have been observed with these new methods and, inevitably, analytic errors also change.^20^^,^^21^

The survey protocol agreed by the Ministry of Health, Labour, and Welfare in Japan and SRL stipulates that the same analytic system for the NHNS (BioMajesty 8060 device No. 1, JEOL Ltd.; installed in the SRL Medical Ultimate Quality Service [MUQS] Laboratory) should also be used for blood examinations that are independently entrusted by prefectures to SRL. This protocol allows PHNS and NHNS results to be monitored in the same manner and permits PHNS data to be added to NHNS. The sample numbers of the PHNS are generally larger than those of the NHNS. However, there are 2 limitations in the use of PHNS data: the measured items differ according to prefecture, and it is possible that the analytic laboratory was changed from SRL to a local laboratory or from a local laboratory to SRL. Therefore, before using PHNS results as additional data, the laboratory responsible for the results should be confirmed. In this study, only samples measured by SRL were included.

In this study, on the basis of quality control results, target TE values for the subsequent 5 years were determined. Specifically, the acceptable limit was defined as the upper 80% confidence limit of TE. TE values above this limit were considered to be in the borderline or unacceptable range, and a caution was issued. The probability of including borderline or unacceptable ranges using these target values remains at 10% even if performance remains equal to that during the previous 12-year period. Assuming annual improvements in performance, approximately 50% of TE values in the subsequent 5-year period are expected to be within the acceptable range. In quality control, there are no absolute criteria for quality, and quality is improved by daily efforts to repeatedly establish and meet criteria. Our monitoring system uses past data to establish target values for a subsequent 5-year period, and adjustments are made by revising target values at 5-year intervals. The system is thus compatible with the idea of quality control. The TE limit for the acceptable and borderline ranges was established for monitoring during 2011–2015, not for its application to past data. Application to the year 2011 (Table 2) confirms the suitability of the proposed TE criteria. When TE falls within the acceptable or borderline ranges, annual continuity and comparability of survey results can be regarded as satisfactory. However, when TE falls within the unacceptable range, measurement values should be used with caution.

Precision is an index of the reproducibility of measurement values obtained by a laboratory. In this study, since TE was calculated using an equation, CV was limited to a singlicate value (*n* = 1) in internal quality control sera for 20 days. CV was calculated from 2 types of commercially available internal quality control serum in SRL. However, if there was a difference of 10% or more in CV between the concentrations of internal quality control materials, the higher CV was used.^7^

In lipid standardization by CDC/CRMLN,^12^ the accuracy, precision, and TE for SRL measurements of TC, HDL-C, and LDL-C met CDC criteria (Table 3) for clinical laboratory use. Therefore, concerning these 3 lipid items, all results in the NHNS and the results in some PHNS can be compared with results in Western countries. However, only results obtained during the previous 9-year period are available for LDL-C, and it is desirable to use these results as a reference.

In conclusion, we used TE criteria to develop a revised 3-level assessment of test performance and evaluated the continuity and comparability of 14 blood chemistry items assayed at SRL for the NHNS and PHNS in Japan. To further improve reliability, TE performance criteria should be updated every 5 years.

## ONLINE ONLY MATERIALS

The Japanese-language abstract for articles can be accessed by clicking on the tab labeled Supplementary materials at the journal website http://dx.doi.org/10.2188/jea.JE20120032.

Abstract in Japanese.
