# Supplementary material for: Revised System to Evaluate Measurement of Blood Chemistry Data From the Japanese National Health and Nutrition Survey and Prefectural Health and Nutrition Surveys
Source: J Epidemiol. 2013 Jan 5;23(1):28–34. doi: 10.2188/jea.JE20120032 (PMC3700228; doi:10.2188/jea.JE20120032)
Supplement: Abstract in Japanese. [file je-23-028-s001.pdf]

# わが国の国民健康・栄養調査と都道府県民健康・栄養調査における血液化学検査成績を評価するためのモニタリング・システム（改訂版）

中村雅一[1]、木山昌彦[2]、北村明彦[2]、石川善紀[2]、佐藤眞一[3]、野田博之[4,5]、吉池信男[6]

[1]国立循環器病研究センター予防健診部、[2]大阪府立成人病センター集団検診第Ⅰ部、[3]千葉県衛生研究所、[4]大阪大学大学院医学系研究科医療経済産業政策学、[5]厚生労働省健康局がん対策・健康増進課、[6]青森県立保健大学健康科学部

【背景】わが国の国民健康・栄養調査と都道府県民健康・栄養調査で集計される血液化学検査成績を総合誤差の大きさから評価・判別するためのモニタリング・システムを再構築した。

【方法】国民健康・栄養調査の全部と一部の都道府県民健康・栄養調査の血液検体は、㈱SRL（東京都八王子市）で同一装置・同一精度管理体制のもとに委託分析されている。1999年から2010年までの10年間における血液化学検査14項目を対象に、SRLの外部精度管理成績から正確度を、内部精度管理成績から精密度を求め、2つの指標から総合誤差(Total Error、%)を計算した。総合誤差の許容範囲は10年間の中央値の信頼限界の80%以下に、非許容範囲はその2倍の値と規定し、許容範囲と非許容範囲の中間域をﾎﾞｰﾀﾞｰﾗｲﾝと規定した。判定は、血液化学検査項目の総合誤差が許容範囲に入る場合は使用可、ﾎﾞｰﾀﾞｰﾗｲﾝに入る場合は注意して使用可、非許容範囲に入る場合は利用することは望ましくないとする。

【結果】計算の結果、ﾎﾞｰﾀﾞｰﾗｲﾝを含む総合誤差の上限値は次の値となった。総コレステロール 5.7%、HDL コレステロール 9.9%、LDL コレステロール 10.0%、トリグリセライド 10.4%、総タンパク 6.6%、アルブミン 7.6%、クレアチニン 10.8%、ブドウ糖 6.5%、γ-GTP 9.7%、尿酸 9.7%、尿酸 7.7%、尿素窒素 8.7%、GOT 9.2%、GPT 9.5%、HbA1c 6.5%であった。

【結論】医学研究者が、わが国の国民健康・栄養調査と都道府県民健康・栄養調査で集計された14項目の血液化学検査成績を利用して、その項目の経年的な継続性や地域比較の可能性を検討するに際し、非許容範囲に入る成績を使うことによって予期しない錯誤や誤った結論に到達するリスクを避けるために、総合誤差による3レベル（Acceptable、Borderline、Unacceptable）のモニタリング・システムを再構築した。

キーワード：モニタリング・システム、正確度、精密度、総合誤差
